# Traumatic Cervical Nerve Root Avulsion with Pseudomeningocele Formation

**DOI:** 10.7759/cureus.1028

**Published:** 2017-02-14

**Authors:** Ali S Haider, Ian T Watson, Suraj Sulhan, Dean Leonard, Eliel N Arrey, Umair Khan, Phu Nguyen, Kennith F Layton

**Affiliations:** 1 Texas A&M College of Medicine; 2 UT Houston Medical School, Memorial Hermann; 3 School of Medicine, St. Georges University; 4 Department of Radiology, Baylor University Medical Center

**Keywords:** cervical nerve root avulsion, epidural hematoma, pseudomeningocele

## Abstract

Cervical nerve root avulsion is a well-documented result of motor vehicle collision (MVC), especially when occurring at high velocities. These avulsions are commonly traction injuries of nerve roots that may be accompanied by a tear in the meninges through the vertebral foramina with associated collections of cerebrospinal fluid (CSF), thereby resulting in a pseudomeningocele. We present a case of a 19-year-old male who experienced an MVC and was brought to the emergency department (ED) with right arm paralysis and other injuries. A neurological examination demonstrated intact sensation but 0/5 muscle strength in the right upper extremity. A magnetic resonance imaging (MRI) of the spinal cord demonstrated massive epidural hematomas extending the length of the cervical spine caudally from C2. An MRI of the right brachial plexus showed C3-C7 anterior horn cell edema and associated traumatic nerve root avulsion with pseudomeningoceles on the right from C5-C8. The development of spinal cord hematoma with these injuries has rarely been documented in the literature and the multiple level avulsion described here with extensive hematoma is a rare clinical presentation. A literature review was conducted to determine the diagnostic requirements, treatment strategies, and complications of such an injury. Our patient received conservative treatment of the right brachial plexus injury and was transferred to an inpatient rehabilitation facility 13 days later.

## Introduction

Nerve root avulsion is a severe form of nerve root injury characterized by a complete tear of one or more of the spinal nerve roots. Avulsion injuries are commonly associated with impact or traction caused by high-energy trauma during a motor vehicle collision (MVC) [1-2]. Pseudomeningocele formation occurs subsequent to avulsion injury due to an accumulation of cerebrospinal fluid (CSF) in the collaterally damaged meninges surrounding damaged nerve roots [3]. Hematoma formation and resultant mass effect are less common complications [4]. Neurological deficits associated with nerve root avulsion range from partial motor function loss to complete paralysis and may be repaired surgically [1,5]. Magnetic resonance imaging (MRI), physical exam, and nerve conduction studies are used in combination to localize the injury and determine the extent of neurological deficits [6]. Here, we present a rare and interesting case of nerve root avulsion with pseudomeningocele and epidural hematoma formation following an MVC. Informed consent was obained from the patient for this study.

## Case presentation

**Figure 1:**
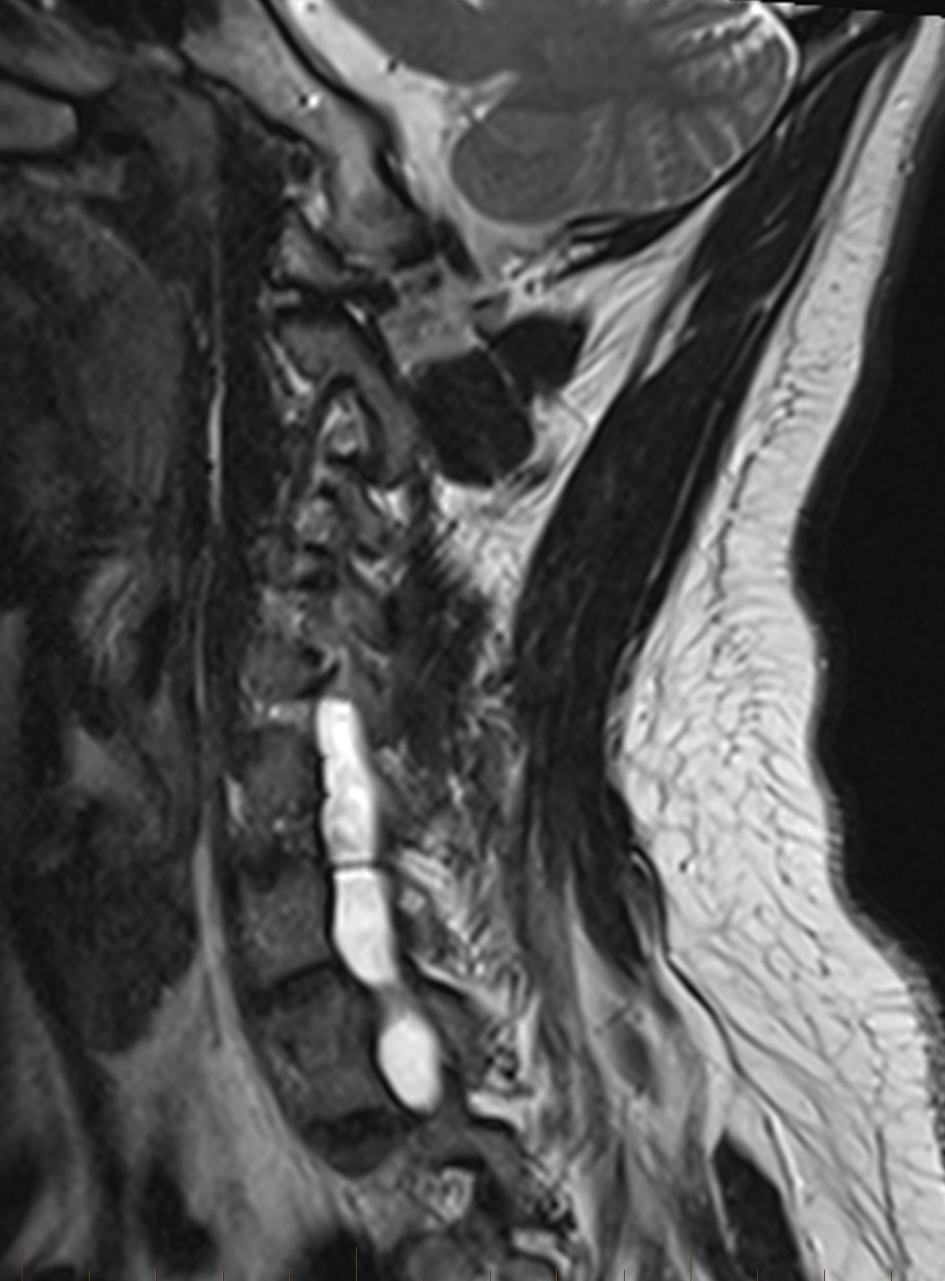
Sagittal right side of cervical spine. Sagittal T2 sequence through the right side of the cervical spine demonstrates the hyperintense and enlarged foramina consistent with pseudomeningoceles in the lower cervical spine. There are no nerve roots exiting the foramina at the involved levels.

**Figure 2:**
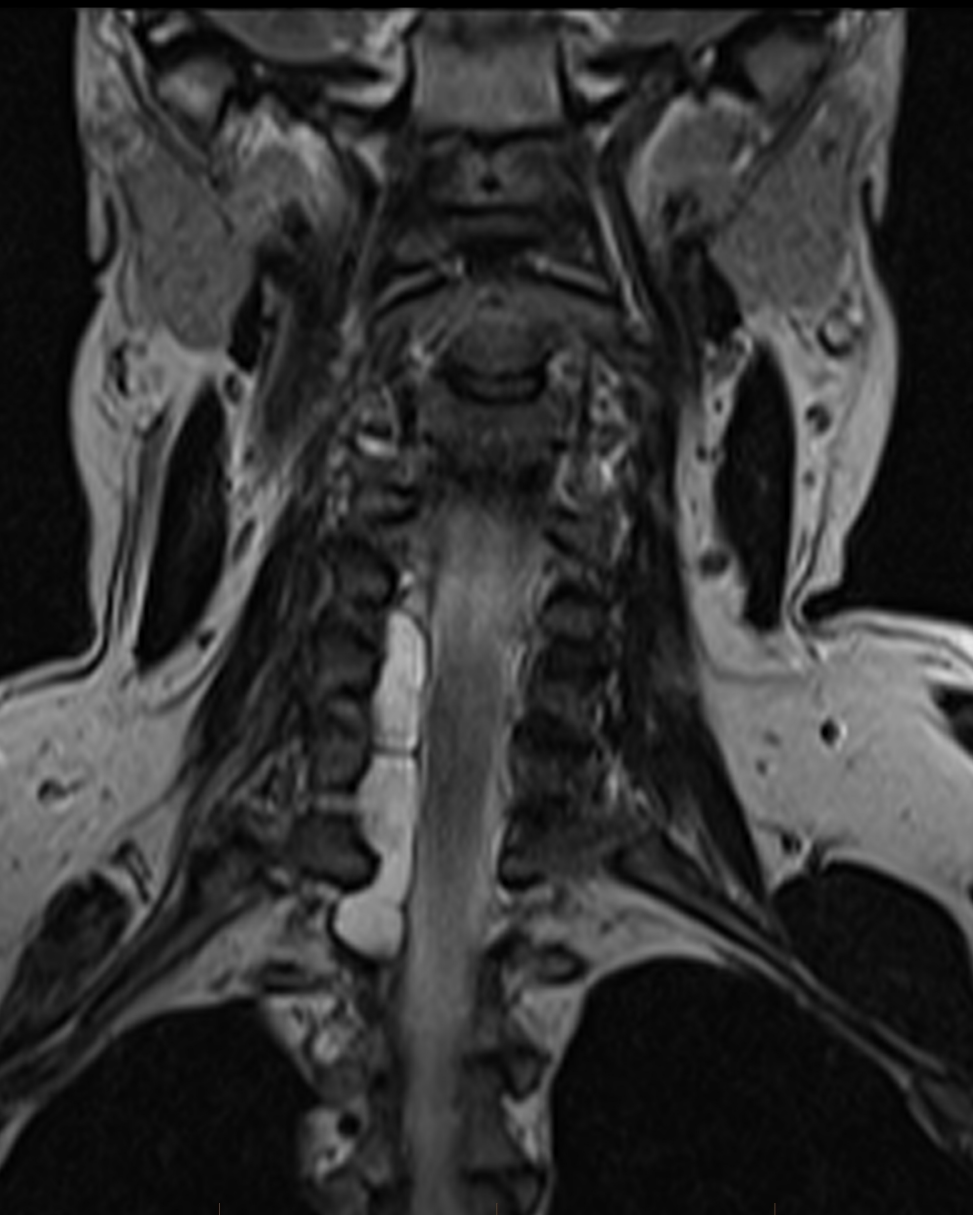
Coronal cervical spine. Coronal T2 sequence again demonstrates hyperintense signal in the right aspect of the cervical spine, causing deviation of the cord to the left and remodeling of multiple right-sided neural foramina.

**Figure 3:**
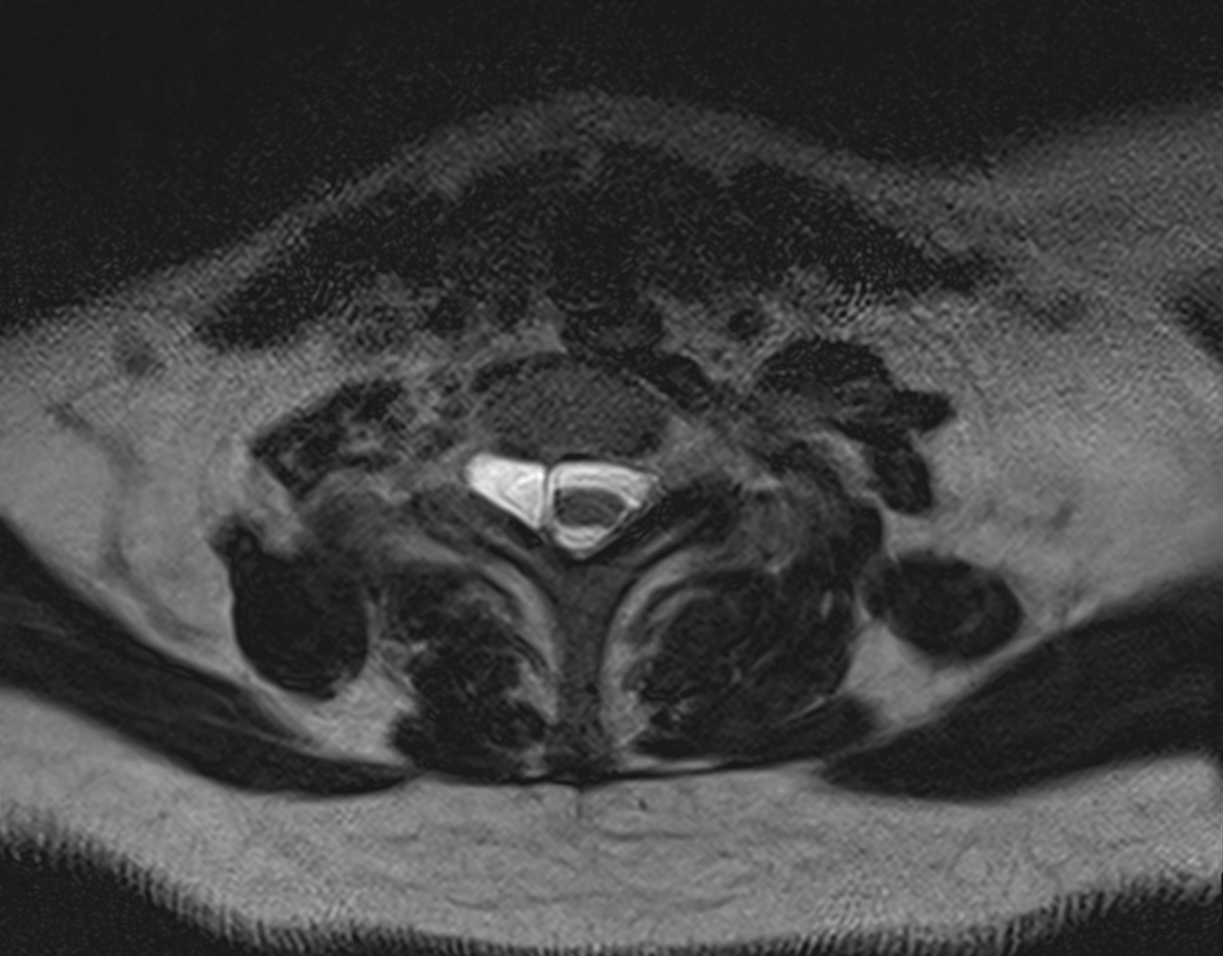
Right-sided pseudomeningocele. Axial T2 sequence reveals the right-sided pseudomeningocele manifested as a T2 hyperintense signal, similar to CSF, between the spinal cord and right neural foramen. Note the long-standing enlargement of the right neural foramen.

We present a case of a 19-year-old male who presented following an MVC with a somatic dysfunction classification of TART (tissue texture change, asymmetry, restriction, and tenderness) II. He had sustained multiple injuries, including right femur fracture, left pubic symphysis fracture, bilateral rib fractures with trace pneumothoraces, and right arm paralysis. The patient was stabilized following admission and a neurologic consult was requested. The neurological examination demonstrated 0/5 muscle strength in all muscle groups of the right arm with intact sensation and 2+ pulses throughout. Both computerized tomography (CT) and MRI of the cervical vertebrae and right brachial plexus without contrast showed no spinal fractures but demonstrated empty nerve root sleeves of the right C5-C8 levels, suggesting traumatic avulsive injury with the development of pseudomeningoceles (Figures 1-3). The patient also suffered from a right anterior epidural hematoma from C2-T4 with posterior displacement of the right hemicord and thecal sac effacement. A dorsal epidural hematoma was also noted from the T1 level caudally out of view of the cervical MRI. A subsequent thoracic spine MRI with gadolinium contrast demonstrated that the dorsal hematoma caused ventral cord displacement from T3-T11 with complete thecal sac effacement from T4-T9, then tapered caudally out of view of the MRI images without further mass effect. Despite the significant mass of the patient's epidural hematomas and the forces they presented on the spinal cord, the patient had no additional complaints of loss of sensation, motor control, or incontinence. As such, it was determined that the most appropriate action would be the conservative management of the epidural hematomas. A Physical Medicine and Rehabilitation consult was ordered to assess the extent of his injuries, and his right upper extremity was placed in a brace. The patient remained stable in the intensive care unit and was eventually transferred to an inpatient rehabilitation facility for further management.

## Discussion

Nerve root avulsion is a devastating injury that can lead to lifelong neurological deficits including complete limb paralysis. The causes of injury include sports accidents, MVCs, falls, gunshot wounds, and the use of forceps at birth. Pseudomeningocele formation is a well-documented phenomenon associated with avulsion injuries. Surgical management aims to transfer peripheral nerve grafts, yielding limited results [7]. Bertelli and Ghizoni demonstrated the recovery of deficits in six of eight surgically managed patients and emphasized rapid intervention, considering time-related neuronal loss [7]. However, the recovery noted in these patients was located purely in the proximal muscle groups; the level of recovery was limited and required direct implantation into the spinal cord [7].

Spinal epidural hematoma (SEDH) is an uncommon manifestation of traumatic nerve root avulsion, accounting for less than one percent of all spinal injuries [8]. It is suspected that the blood is venous in origin due to the richly vascularized venous plexus surrounding the spinal cord [9]. SEDH may have a spontaneous etiology, although vertebral fracture, birth trauma, lumbar puncture, and surgical complications are acute causes. Cervical spondylosis, rheumatoid arthritis, Paget’s disease, and ankylosing spondylitis are thought to be predisposing factors for this condition [8-9]. SEDH is considered a medical emergency due to the complications associated with the mass effect and long-term deficits, requiring prompt MRI and possibly surgical intervention [8,10]. Time to surgery and degree of preoperative deficits are the best prognostic indicators when considering patient outcomes [9-10]. An analysis, by Foo and Rossier, of 154 patients with spinal epidural hematoma stratified patients by time to intervention and found that surgical decompression within 36 hours was associated with complete recovery in 33% of patients and partial recovery in 46% of patients [9]. However, when the patient's neurologic course is stable or improving, surgical intervention is not necessarily indicated and conservative management of the SEDH may be initiated [7]. Our patient presented with two cervicothoracic SEDHs that were subsequent to nerve root avulsion and pseudomeningocele formation. This constellation of findings was particularly unique and is a reminder to consider these rare complications in the diagnosis and management of avulsion injuries.

## Conclusions

Traumatic brachial nerve root avulsion with pseudomeningocele formation is a devastating injury that, in the case presented, produced permanent paralysis of the right upper extremity. Though nerve grafts are a promising route of treatment of avulsive injury in the future, significant research into the biomechanics of nerve grafting and improvements in surgical technique are required. Further, the avulsive injury to four nerve roots of the brachial plexus was complicated by spinal epidural hematoma formation and mass effect on the spinal cord throughout the cervical and thoracic regions. Venous blood is thought to be the source of hematoma formation, owing to the intimal involvement of the spinal venous plexus. Preoperative deficits and time to surgery are the best prognostic indicators associated with hematoma formation when there is an evidence of compressive myelopathy. Though our case illustrated two asymptomatic spinal epidural hematomas that were treated conservatively due to the absence of compressive myelopathy symptoms, the case details the importance of identifying and treating such a potentially reversible cause of permanent neurological damage. 

## References

[1] Carlstedt T (2009). Nerve root replantation. Neurosurg Clin N Am.

[2] Faglioni W Jr, Siqueira MG, Martins RS, Heise CO, Foroni L (2014). The epidemiology of adult traumatic brachial plexus lesions in a large metropolis. Acta Neurochir (Wien).

[3] Freedy RM, Miller KD Jr, Eick JJ, Granke DS (1989). Traumatic lumbosacral nerve root avulsion: evaluation by MR imaging. J Comput Assist Tomogr.

[4] Newman WC, Tempel ZJ, Tyler-Kabara EC (2015). Posttraumatic cervical nerve root avulsion with epidural hematoma. World Neurosurg.

[5] Thatte MR, Babhulkar S, Hiremath A (2013). Brachial plexus injury in adults: diagnosis and surgical treatment strategies. Ann Indian Acad Neurol.

[6] Caporrino FA, Moreira L, Moraes VY, Belloti JC, Gomes dos Santos JB, Faloppa F (2014). Brachial plexus injuries: diagnosis performance and reliability of everyday tools. Hand Surg.

[7] Bertelli JA, Ghizoni MF (2003). Brachial plexus avulsion injury repairs with nerve transfers and nerve grafts directly implanted into the spinal cord yield partial recovery of shoulder and elbow movements. Neurosurgery.

[8] Garg K, Satyarthee GD, Singla R, Sharma BS (2014). Extensive long-segment cervicothoracic traumatic spinal epidural hematoma with avulsion of C7, C8, and T1 nerve roots. J Neurosci Rural Pract.

[9] Foo D, Rossier AB (1982). Post-traumatic spinal epidural hematoma. Neurosurgery.

[10] Mukerji N, Todd N (2013). Spinal epidural haematoma; factors influencing outcome. Br J Neurosurg.

